# Assessment of the Extent of Resection in Surgery of High-Grade Glioma—Evaluation of Black Blood Sequences for Intraoperative Magnetic Resonance Imaging at 3 Tesla

**DOI:** 10.3390/cancers12061580

**Published:** 2020-06-15

**Authors:** Tom Finck, Jens Gempt, Sandro M. Krieg, Bernhard Meyer, Claus Zimmer, Benedikt Wiestler, Jan S. Kirschke, Nico Sollmann

**Affiliations:** 1 Department of Diagnostic and Interventional Neuroradiology, Klinikum rechts der Isar, Technische Universität München, Ismaninger Str. 22, 81675 Munich, Germany; Tom.Finck@tum.de (T.F.); Claus.Zimmer@tum.de (C.Z.); B.Wiestler@tum.de (B.W.); Jan.Kirschke@tum.de (J.S.K.); 2 Department of Neurosurgery, Klinikum rechts der Isar, Technische Universität München, Ismaninger Str. 22, 81675 Munich, Germany; Jens.Gempt@tum.de (J.G.); Sandro.Krieg@tum.de (S.M.K.); Bernhard.Meyer@tum.de (B.M.); 3 TUM-Neuroimaging Center, Klinikum rechts der Isar, Technische Universität München, 81675 Munich, Germany

**Keywords:** advanced imaging, brain tumor, contrast enhancement, extent of resection, high-grade glioma, intraoperative magnetic resonance imaging, neurosurgery, tumor residual

## Abstract

Achieving an optimal extent of resection (EOR) whilst keeping lasting neurological decline to a minimum is paramount for modern neurosurgery in patients with high-grade glioma (HGG). To improve EOR assessment, this study introduces Black Blood (BB) imaging, which uses a selective saturation pulse to suppress the blood signal, to 3-Tesla intraoperative magnetic resonance imaging (iMRI). Seventy-three patients (56.4 ± 13.9 years, 64.4% male) with contrast-enhancing HGGs underwent iMRI, including contrast-enhanced (CE) and non-CE 3D turbo field-echo imaging (TFE; acquisition time: 4:20 min per sequence) and CE and non-CE 3D BB imaging (acquisition time: 1:36 min per sequence). Two readers (R1 and R2) retrospectively evaluated the EOR and diagnostic confidence (1—very inconfident to 5—very confident) as well as the delineation of tumor boarders and spread of contrast-enhancing tumor components (in case of contrast-enhancing tumor residuals). Furthermore, the contrast-to-noise ratio (CNR) was measured for contrast-enhancing tumor residuals. Both BB and conventional TFE imaging allowed for the correct detection of all contrast-enhancing tumor residuals intraoperatively (considering postsurgical MRI and histopathological evaluation as the ground truth for determination of the lack/presence of contrast-enhancing tumor residuals), but BB imaging showed significantly higher diagnostic confidence (R1: 4.65 ± 0.53 vs. 3.88 ± 1.02, *p* < 0.0001; R2: 4.75 ± 0.50 vs. 4.25 ± 0.81, *p* < 0.0001). Delineation of contrast-enhancing tumor residuals and detection of their spread into adjacent brain parenchyma was better for BB imaging. Accordingly, significantly higher CNRs were noted for BB imaging (48.1 ± 32.1 vs. 24.4 ± 15.3, *p* < 0.0001). In conclusion, BB imaging is not inferior to conventional TFE imaging for EOR assessment, but may significantly reduce scanning time for iMRI whilst increasing diagnostic confidence. Furthermore, given the better depiction of contrast-enhancing tumor residual spread and borders, BB imaging could support achieving complete macroscopic resection in patients suffering from HGG, which is clinically relevant as an optimal EOR is correlated to prolonged survival.

## 1. Introduction

Although primary tumors of the central nervous system account for only a low percentage among all malignancies in humans, high-grade gliomas (HGGs) with their most common subtypes anaplastic astrocytoma and glioblastoma multiforme have an exceedingly high mortality with median survival rates in the range of few months [1,2,3,4]. While adjuvant tumor border radiation combined with chemotherapy is considered standard of therapy, debulking surgery remains the first-line treatment in HGGs. Many studies have thus been able to demonstrate that maximizing the extent of resection (EOR) in HGG surgery cannot only extent progression-free survival (PFS) but also enhance quality of life [5,6,7,8,9].

Given the association of an optimal EOR with prolonged survival as well as favorable functional outcome, the efforts to reach complete tumor resection, ideally without causing any lasting neurological deficits, have been manifold. The gold standard to map functionally eloquent structures is intraoperative direct electrical stimulation (DES), which can be effectively combined with intraoperative neuromonitoring to reduce the risk of damaging eloquent structures [10,11,12,13]. Furthermore, resection control via intraoperative magnetic resonance imaging (iMRI) has its merits since intraoperative detection of remnant tumor tissue may allow to increase EOR rates as debulking surgery could directly continue after iMRI acquisition.

Technical advances over the last decades enabled scanning at high magnetic fields in justifiable time, using mostly multi-sequence protocols for intraoperative assessment of the brain. Specifically, iMRI may help to master the onco-functional balance dictated by the strive to ideally remove the entire tumor mass whilst avoiding surgery-related functional decline, which reflects the principle of modern neuro-surgical oncology [14,15]. Indeed, iMRI demonstrated high potential to increase the overall EOR and related survival and to reduce perioperative morbidity [16,17,18,19]. The EOR was increased from 76% to 96% in a prospective study analyzing the utility of contrast-enhanced (CE) T1-weighted spin-echo sequences acquired by iMRI at 1.5 Tesla in patients with primary brain tumors [17]. Another study was able to report a doubling in PFS from 6.6 to 12.5 months after evaluating magnetization-prepared rapid gradient-echo (MP-RAGE) sequences from a 1.5-Tesla iMRI machine during resections of glioblastomas in eloquent regions [19].

However, sequence developments are still primarily restricted to the preoperative MRI acquisition, and one has to consider what implementations could further increase the utility of iMRI and consolidate its role as a standard tool in neuro-oncological surgery. On this note, improved depiction of tumor-related contrast enhancement—the hallmark of the impaired blood-brain barrier generally accompanying HGGs—on iMRI using advanced T1-weighted sequences might be achieved. This is particularly relevant during tumor resection since blood leakage, clotting, or perioperative anatomical derangement can hamper timely and accurate discrimination of residual tumor tissue. Recent advances in neuroradiological imaging hint at the superiority of Black Blood (BB) sequences compared to more established T1-weighted sequences (e.g., MP-RAGE) in depicting various types of contrast-enhancing pathologies [20,21]. Specifically, in a heterogeneous pool of intracranial malignomas comprising primary brain tumors and metastases, CE BB sequences allowed to detect smaller lesions and metastases that would otherwise have been missed with T1-weighted gradient-echo imaging [20]. Similarly, CE BB sequences outperformed both CE T1-weighted gradient- and spin-echo sequences in depicting leptomeningeal carcinomatosis at 3 Tesla [21].

Given that blood residues adjacent to the resection zone can render definition of the EOR troublesome on conventional T1-weighted sequences, BB imaging, which uses a selective saturation pulse to suppress the blood signal, could facilitate detecting contrast-enhancing residual tumor tissue during iMRI. Our hypothesis is that based on these intrinsic properties, contrast-enhancing tumor residuals can be better identified and, thus, BB imaging may increase the diagnostic confidence in interpreting iMRI during resection of HGGs. We therefore aim to investigate the utility of BB imaging during iMRI among patients with HGGs and compare it to established, standardly used T1-weighted turbo field-echo (TFE) imaging at 3 Tesla. 

## 2. Materials and Methods

### 2.1. Study Protocol

This study represents a retrospective analysis of prospectively collected monocentric data. Approval of the Institutional Review Board (registration number: 340/16 S) and written informed patient consent were obtained from all patients for the prospective data collection.

Intraoperative acquisition of BB imaging was performed in the setting of clinical routine after an update of the iMRI scanning protocol for glioma patients in early 2019. For patient inclusion, the hospital-intern Picture Archiving and Communication System (PACS) was retrospectively searched for eligible patients from March 2019 to February 2020 considering the following inclusion criteria: 1) availability of iMRI in the setting of neurosurgical resection of a primary or recurrent HGG (as confirmed by either biopsies or final histopathological evaluation), 2) acquisition of a TFE and BB sequence before and after administration of a contrast agent in the same iMRI session (as part of a standardized tumor imaging protocol), 3) availability of postsurgical MRI (to serve as ground truth for determination of the lack/presence of contrast-enhancing tumor residuals, together with histopathological evaluation), and 4) age above 18 years. The following exclusion criteria were defined: 1) non-contrast-enhancing HGG (as indicated by preoperative MRI), and 2) artifacts in imaging data not allowing for diagnostic use.

### 2.2. Intraoperative Magnetic Resonance Imaging

Images were acquired intraoperatively following a standardized, institute-specific multi-sequence protocol that remained the same during the time interval of study inclusion. A 3-Tesla scanner (Philips Ingenia; Philips Healthcare, Best, The Netherlands) with an eight-channel head coil (OR Head Coil; NORAS MRI Products GmbH, Höchberg, Germany) was used for image acquisition. Patients were scanned in a supine position, under general anesthesia, after having been transferred from the operating theater to the iMRI examination room that was located directly adjacent.

Identical parameters were used in all patients for TFE imaging (repetition time [TR] = 9 ms, echo time [TE] = 4 ms, TFE factor = 195, 1 TFE inversion prepulse at 1000 ms, flip angle = 8°, acquisition mode: 3D, acquisition duration: 4:20 min, acquired in the sagittal plane with an isotropic voxel size of 1 mm^3^) and turbo spin-echo (TSE) BB imaging (TR = 4000 ms, TE = 35 ms, echo train length = 55, no inversion prepulse, flip angle = 90°, acquisition mode: 3D, acquisition duration: 1:36 min, acquired in the sagittal plane with an isotropic voxel size of 1 mm^3^), with acquisitions taking place before and after intravenous contrast administration. For both TFE and BB imaging, parallel imaging using Compressed SENSE was used (acceleration factor of 3.5 for TFE and 8 for BB sequences). A dose of 0.2 mL per kg body weight of gadoteric acid (DOTAGRAF^®^ 0.5 mmol/mL, Jenapharm GmbH, Jena, Germany) was administered automatically via a pressure pump, with a delay of 4:27 min between administration and acquisition of CE TFE sequences and 8:47 min between administration and acquisition of CE BB sequences.

In case of contrast-enhancing residual tumors on iMRI, it was the neurosurgeon’s decision to end the procedure or continue resection based on situs inspection and the interpretation of imaging data together with the neuroradiologists. Determination of a contrast-enhancing tumor residual intraoperatively during clinical routine was based on analysis of both TFE and BB imaging.

### 2.3. Image Analysis

For this study, two neuroradiologists with 3 years (reader 1 = R1) and 7 years of experience (reader 2 = R2) in interpretation of neuroradiological imaging data retrospectively analyzed the images derived from iMRI in the PACS viewer (IDS7; Sectra AB, Linköping, Sweden) regarding the EOR and diagnostic confidence as well as features of qualitative image assessment. Additional quantitative evaluation of contrast-enhancing residual tumors was performed by R1.

The readers were blinded to all patient-related information and radiological reports, to the scorings of each other, to further sequences acquired intraoperatively (except for the TFE and BB sequences before and after contrast administration), and to preoperative and follow-up MRI as performed during clinical routine. The readers were furthermore blinded regarding the performance of further resection due to suspected residual tumor tissue on iMRI in clinical routine, but could not be reliably blinded to the sequence type (TFE or BB sequences) due to the distinct appearance of both sequences. The order of patient cases was subject to randomization. The readers were allowed to use axial, sagittal, and coronal slices and to manually adapt image contrast at the PACS working stations.

#### 2.3.1. Extent of Resection and Diagnostic Confidence

Complete resection of the initially contrast-enhancing tumor tissue was defined as entire removal of tumor-related contrast enhancement, while incomplete resection was noted if remaining tumor-related contrast enhancement was present on iMRI. The determination of complete or incomplete resection of contrast-enhancing tumor tissue on iMRI was done separately for CE TFE and CE BB sequences, with non-CE TFE and non-CE BB sequences being available in co-registered mode. Correlation with postoperative MRI (if no secondary resection during the same surgery after iMRI took place) and, additionally, verification by the histopathology report (lack/presence of tumor in tissue probes if secondary resection took place) served as the ground truth for alleged contrast-enhancing tumor residuals.

Remaining contrast-enhancing tumor tissue on iMRI was categorized as either expected if it was in functionally eloquent brain areas (and the non-willingness to achieve complete resection was stated by the senior neurosurgeon) or presented in the form of satellite lesions distant from the surgery area. Moreover, it was considered unexpected if the senior neurosurgeon reported that gross total resection (determined based on intraoperative situs inspection and the interpretation of CE and non-CE imaging) was achieved. Beyond the binary evaluation if contrast-enhancing tumor residuals were depicted in CE TFE or CE BB sequences, readers determined their diagnostic confidence for reporting any tumor residuals on iMRI (1—very inconfident, 2—weakly inconfident, 3—undecided, 4—weakly confident, and 5—very confident).

#### 2.3.2. Qualitative Image Assessment

Overall image quality was rated on a 5-point Likert scale (1—bad, 2—medium to bad, 3—medium, 4—good, and 5—very good image quality). Furthermore, for each contrast-enhancing tumor residual on iMRI, determination was made if the spreading into the adjacent brain parenchyma, i.e., the extent of contrast enhancement as seen on CE TFE or CE BB sequences, was comparable or greater in one of both investigated sequences (more extensive in CE TFE, more extensive in CE BB, or same extents in both sequences). Delineation of contrast-enhancing tumor residuals to adjacent brain parenchyma, i.e., the differentiability of contrast-enhancing tumor borders from surrounding brain parenchyma, was rated on a 3-point Likert scale (1—bad, 2—intermediate, and 3—good delineation/conspicuity).

#### 2.3.3. Quantitative Image Assessment

For quantitative assessment, manual volumetry of contrast-enhancing tumor residuals on iMRI was performed using an open-source software (ITK-Snap, version 3.8.0) [22]. Furthermore, the contrast-to-noise ratio (CNR) was estimated as follows for CE TFE and CE BB sequences, similar to previous work [20]:

CNR = (SI_Lesion_ − SI_NAWM_)/SD_NAWM_

To calculate the CNR, a representative region of interest (ROI) was manually drawn in the contrast-enhancing tumor residual, with an equally-sized ROI being placed in the normal-appearing white matter (NAWM) of the contralateral hemisphere. The signal intensity (SI; the average signal intensity of the respective ROI) and the standard deviation (SD; the average standard deviation of the respective ROI) were extracted for each case. The areal size of the ROI was individually adapted to not include brain parenchyma surrounding the contrast-enhancing residual tumor tissue, and a margin was left to not include ultimate tumor-parenchyma border zones.

### 2.4. Statistical Analysis

GraphPad Prism (version 8.3.1; GraphPad Software Inc., La Jolla, CA, USA) was used for statistical analysis. Descriptive statistics including relative or absolute frequencies and mean ± SD were calculated. Shapiro-Wilk normality test indicated non-Gaussian data distribution for the majority of assessed parameters. *p*-values < 0.05 were considered statistically significant.

Wilcoxon matched-pairs signed rank tests were used for comparing scoring for CE TFE versus CE BB sequences for R1 and R2, respectively, considering scores assigned for image quality, diagnostic confidence, and delineation of contrast-enhancing tumor to surrounding brain parenchyma. The tumor volumes of contrast-enhancing residuals and CNRs on iMRI were also compared with Wilcoxon matched-pairs signed rank tests between measurements performed in CE TFE versus CE BB sequences. Correction for multiple testing was applied using the Benjamini-Hochberg procedure with a false discovery rate of 10% [23].

Sensitivity and specificity of CE TFE and CE BB sequences to depict remaining contrast-enhancing tumor tissue were calculated based on the ratings from R1 and R2 and ground truths for tumor residuals.

## 3. Results

### 3.1. Patient Data

A consecutive series of 85 patients that received iMRI and were diagnosed with HGGs according to histopathological evaluation were enrolled. Tumors in 12 patients did not show contrast-enhancement on presurgical MRI and thus needed to be excluded.

The remaining 73 patients (mean age: 56.4 ± 13.9 years, 64.4% male) were analyzed by R1 and R2 under the study’s premises. Of these, 58 patients harbored glioblastoma multiforme (World Health Organization (WHO) grade IV), 12 anaplastic astrocytoma (WHO grade III), 2 anaplastic oligodendroglioma (WHO grade III), and 1 gliosarcoma (WHO grade IV), all presenting with tumor-related contrast enhancement on preoperative MRI.

### 3.2. Surgery and Extent of Resection

Complete resection of contrast-enhancing tumor tissue (for both CE TFE and CE BB sequences) was detected on iMRI in 33 patients (45.2%) according to both readers. These findings were confirmed in 32 patients by absence of any contrast-enhancing tumor tissue on postoperative MRI, with one false-negative count according to the separate evaluation of both readers.

Incomplete resection of contrast-enhancing tumor tissue at the time of iMRI acquisition was detected in 40 of the 73 included patients (54.8%) in both CE BB and CE TFE sequences, with 8 of the 40 tumor residuals (20.0%) being expected due to stop of resection on the basis of functional eloquence or tumor satellites outside of the resection zone. For the remaining 32 cases, additional resections during the same surgery were performed in 25 patients (78.1%) after iMRI acquisition and could verify malignancy of the further resected tissue in 23 of the 25 pathology samples (92.0%). Consequently, follow-up resection in 2 patients (8.0%) did not show unequivocal malignant tissue on histopathological evaluation.

In the 7 remaining cases, tumor tissue was verified on postoperative MRI acquired within the first 48 h after surgery and was therefore considered an unexpected residual. Thus, 66 out of 73 patients (90.4%) showed complete resection on postoperative MRI. With identical findings of one false-negative and three false-positive cases, CE BB and CE TFE sequences had identical values for sensitivity (97.4%) and specificity (91.4%) to detect or exclude tumor residuals on iMRI according to evaluation of both readers.

**Figure 1:**
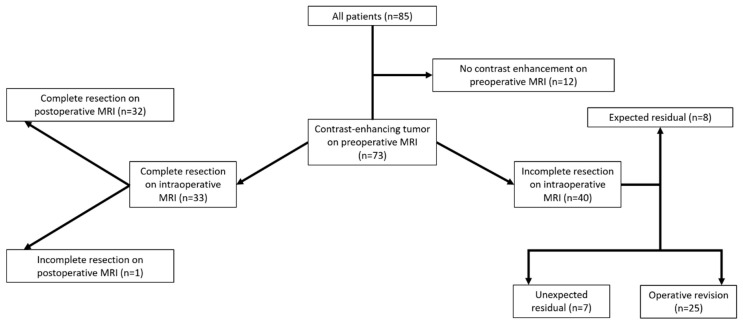
Flow chart for surgical outcome and extent of resection based on evaluation of pre-, intra-, and postoperative imaging.

**Figure 2:**
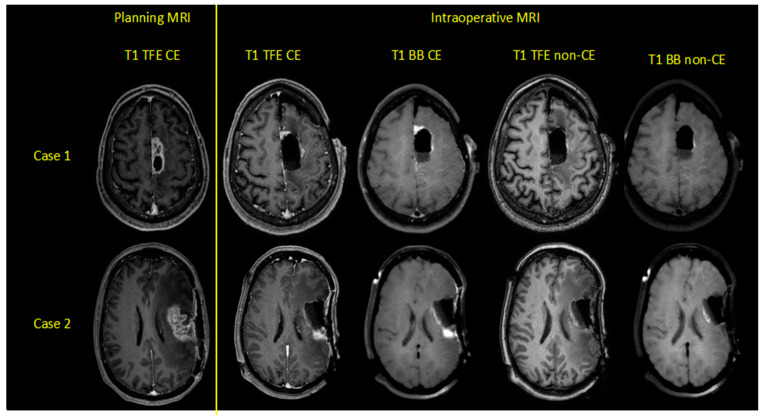
Exemplary cases of two patients harboring left-hemispheric glioblastoma multiforme, depicting contrast-enhanced (CE) and non-CE turbo field-echo (TFE) and Black Blood (BB) sequences for preoperative planning and intraoperative magnetic resonance imaging (iMRI).

A flow chart with the categorization of each patient is provided in Figure 1. Exemplary cases with the investigated iMRI sequences are shown in Figure 2.

### 3.3. Diagnostic Performance of TFE and BB Imaging—Qualitative Analysis

Better subjective image quality in CE TFE versus CE BB sequences was noted by both readers (Table 1). In spite of their identical sensitivity and specificity in detecting contrast-enhancing residual tumor tissue on iMRI, the diagnostic confidence was significantly higher for CE BB compared to CE TFE sequences, irrespective of whether complete or incomplete resection was achieved (Table 1 and Table 2). On average, both readers were very confident in determining the lack/presence of contrast-enhancing tumor residuals when considering CE BB and only weakly confident when considering CE TFE sequences (Table 1 and Table 2).

**Figure 3:**
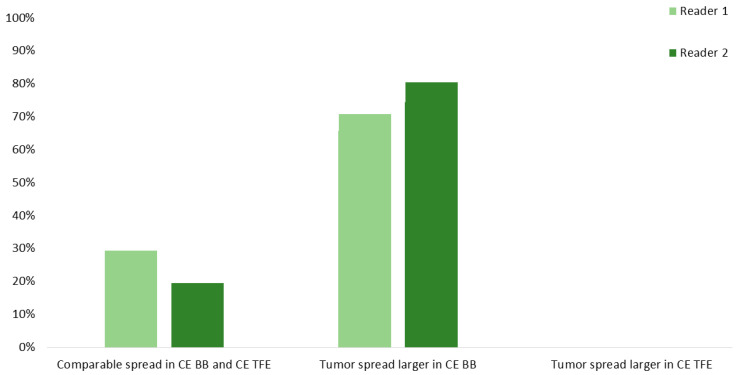
Spread of the contrast-enhancing tumor residuals into adjacent brain parenchyma for contrast-enhanced (CE) turbo field-echo (TFE) and Black Blood (BB) sequences derived from intraoperative magnetic resonance imaging (iMRI). Evaluations were performed by two readers (R1 and R2).

Delineation of tumor residuals against the surrounding parenchyma was significantly improved when considering CE BB instead of CE TFE sequences for R1 (2.83 ± 0.39 vs. 1.95 ± 0.68, *p* < 0.0001) and R2 (2.83 ± 0.39 vs. 2.18 ± 0.71, *p* < 0.0001; Table 2). Specifically, tumor tissue could be delimited sharply with CE BB and only vaguely with CE TFE sequences by both readers. Similarly, tumor spread was subjectively assessed to be vaster in CE BB compared to CE TFE sequences in 28 (70.0%) and 32 (80.0%) cases of the 40 patients with contrast-enhancing tumor residuals for R1 and R2. Similar spread was noted in 12 (30.0%) and 8 (20.0%) cases, respectively, while no patient showed a vaster extent of contrast-enhancing tumor residuals in CE TFE versus CE BB sequences (Figure 3).

### 3.4. Diagnostic Performance of TFE and BB Imaging—Quantitative Analysis

**Figure 4:**
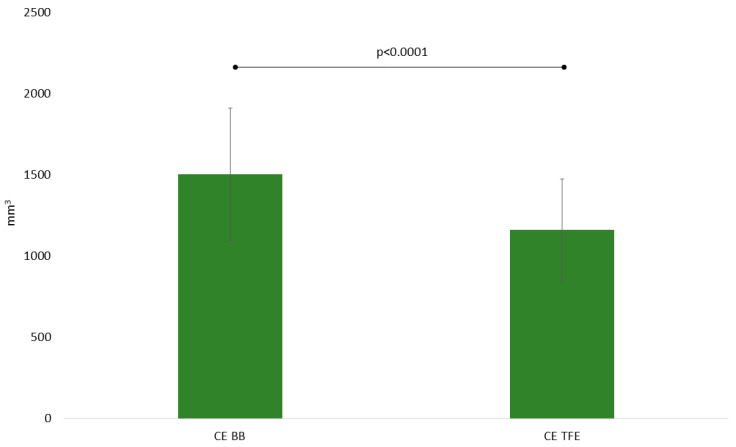
Mean volumes of contrast-enhancing tumor residuals for contrast-enhanced (CE) turbo field-echo (TFE) and Black Blood (BB) sequences as derived from intraoperative magnetic resonance imaging (iMRI).

The above-mentioned subjective assessments regarding tumor spread could be verified by volumetric measurements as contrast-enhancing tumor residuals were significantly larger when considering CE BB (1505 ± 2303 mm^3^) versus CE TFE (1162 ± 1782 mm^3^) sequences (*p* = 0.0010; Table 2, Figure 4). Also, the before-stated better conspicuity of the tumor borders seems in agreement with notably higher CNR in CE BB versus CE TFE sequences (48.1 ± 32.1 vs. 24.4 ± 15.3, *p* < 0.0001; Table 2).

## 4. Discussion

Surgery in HGG is perpetually concerned with maximizing the EOR while minimizing the procedure-linked morbidity in terms of lasting functional decline. In the backlight of this, iMRI has emerged as a promising tool to intraoperatively evaluate the EOR and morphed from a visionary concept in biomedical engineering to an established practice in sophisticated neurosurgical suites worldwide. Utilization of this technique cannot only spare second-look procedures several days after primary surgery in the setting of unexpected incomplete resections; it has also resulted in optimized EOR and prolonged PFS [16,17,18,19].

While the hardware improvements have been impressive in the past decades, 3-Tesla iMRI setups are still not widely distributed, and iMRI-guided surgery in HGG still heavily relies on conventional T1-weighted sequences, such as standard MP-RAGE or TFE sequences [16,17,18,19,24,25]. Several studies have, however, demonstrated the superiority of presaturated T1-weighted sequences such as BB imaging in depicting intracranial pathologies with impaired blood-brain barriers [20,21]. In this context, BB imaging was initially mostly restricted to 2D acquisition mode with only small spatial coverage. However, a new type of TSE BB sequences was introduced with variable flip-angle refocusing pulses, thus enabling 3D imaging with high isotropic resolution [26,27]. Yet, application in HGG imaging is largely lacking, and no previous study used BB imaging for iMRI. As the technique of BB imaging is based on the suppression of blood signal, this approach seems promising in iMRI where orientation on conventional T1-weighted sequences is often arduous due to the proximity of blood deposits with high T1 signal to adjacent tumor residuals.

Although no difference was revealed between the number of detected contrast-enhancing tumor residuals between sequences, our data show that neuroradiologists can provide diagnosis with higher diagnostic confidence when using BB imaging. Explanations for this may include the tidier aspect of the resection area in CE BB sequences as vasculature is suppressed and normal brain parenchyma has a rather dull appearance, leading to notably brighter depiction of contrast-enhancing tumor residuals as confirmed by the superior CNR. Beyond the benefits for patient security as second-look situs inspection could potentially be waived in some cases, this might in total lead to a faster workflow for the team in the operating theater as the acquisition time of a pre- and post-contrast BB sequence (cumulative duration of approximately 3 min) easily dwarfs the duration of a potential second-look procedure or potentially higher image reading times when only conventional T1-weighted sequences are available instead. Particularly in the intraoperative setting, possibilities for reductions in scanning time are welcomed. Furthermore, on the long run, a clearer decision making can improve acceptance of iMRI as a routine tool in HGG surgery. At the current stage we, however, consider the BB technique a complementary sequence and not a replacement for standard T1-weighted sequences—such as TFE imaging—since the clear identification of vasculature remains paramount for intraoperative neuronavigation, which may not be feasible with the newly proposed technique alone.

Of note, our investigation has further shown that vaster spread of contrast-enhancing tumor residuals compared to the established CE TFE sequences could be noted when considering CE BB sequences, which is true not only for subjective assessment, but also for quantitative assessment using basic volumetry. Indeed, it has to be noted that contrast-enhancing tumor volumes appeared approximately 23% (1505 vs. 1162 mm^3^) larger when evaluated with this novel sequence. Given that age-independent survival benefits in glioma patients have been reported up to a threshold of resecting 98% of the tumor volume, such stark differences in depicting malignant tissue on iMRI merit consideration in the clinical setup [28,29,30]. Still, the ability to more generously plan tumor resection is subordinated to the quest to preserve quality of life and neurological function. Hence, an essential priority for the neurosurgeon is to clearly identify tumor borders and, thus, to spare functionally eloquent parenchyma adjacent to the tumor. According to our results, utilization of CE BB sequences allows for sharper identification of tumor boundaries and could consequently help safely manoeuver the resection. Especially in the context of neighboring functionally eloquent structures, such as motor or language pathways, but also in regions with a lot of vasculature, such as the insular region, this feature of CE BB sequences could be helpful to limit morbidity and improve outcome.

Some limitations of the study should be considered. First, the retrospective nature is inferior to a prospective, potentially randomized concept that would be better suited to investigate relevant differences in both techniques with potential ramifications for PFS. Second, the established and standardly used TFE sequences are most widely considered by neurosurgeons to extend resections on the basis of iMRI findings if residuals are present. As brain shift occurs during surgery and a certain, yet difficult to quantify safety margin certainly accompanies these resections, our data does not allow to extrapolate if less unexpected residuals would have been found if BB imaging had been used for surgery planning. Third, the CE TFE sequences were acquired before the CE BB sequences in all patients enrolled in this study by default. Since TFE imaging is considered the current institution-intern standard for T1-weighted imaging on iMRI, it was given priority as reflected by primary acquisition in the scanning protocol. In theory, a later start of imaging acquisition may generally lead to more distinct contrast enhancement characteristics, potentially contributing to higher CNR and clearer depiction of contrast-enhancing residual tumors for CE BB sequences when compared to CE TFE sequences. However, the interval between the acquisition start of both sequences after contrast administration was short and in the range of only few minutes, making it questionable whether this may indeed reflect a source of bias. Nevertheless, future investigations may consider alternating acquisition patterns to confirm the results of this study and to rule out potential bias.

## 5. Conclusions

This study investigated the use of BB imaging for iMRI at 3 Tesla in a consecutive series of 73 patients with HGGs. Implementation of this novel imaging technique for the intraoperative assessment augments the diagnostic confidence to identify the lack/presence of contrast-enhancing tumor residuals, depicts larger volumes of contrast-enhancing residual tumor, and helps to better identify the boundaries of contrast-enhancing malignant tissue. Our findings thus suggest BB imaging to become a reasonable adjunct to iMRI protocols in HGG surgery. Future research may evaluate whether standard T1-weighted sequences, like TFE imaging, could be skipped to markedly reduce acquisition time of iMRI in the clinical neuro-oncological setup.

## Figures and Tables

**Table 1:** Image quality and diagnostic confidence to detect contrast-enhancing tumor residuals for all included patients (*n* = 73 patients).

Item	BB	TFE	*p*-Value
Diagnostic confidence R1	4.16 ± 1.16	3.63 ± 1.17	<0.0001
Diagnostic confidence R2	4.58 ± 0.59	4.14 ± 0.77	<0.0001
Image Quality R1	4.06 ± 0.91	4.43 ± 0.62	0.0002
Image Quality R2	3.75 ± 0.88	4.32 ± 0.57	<0.0001

Evaluations (in *n* = 73 patients) were performed separately for contrast-enhanced (CE) turbo field-echo (TFE) and Black Blood (BB) sequences as derived from intraoperative magnetic resonance imaging (iMRI) by two readers (R1 and R2).

**Table 2:** Delineation, diagnostic confidence, volumes, and contrast-to-noise ratio (CNR) for contrast-enhancing tumor residuals in patients with incomplete resection of contrast-enhancing tumor tissue (*n* = 40 patients).

Item	BB	TFE	*p*-Value
Diagnostic confidence R1	4.65 ± 0.53	3.88 ± 1.02	<0.0001
Diagnostic confidence R2	4.75 ± 0.50	4.25 ± 0.81	<0.0001
Delineation R1	2.83 ± 0.39	1.95 ± 0.68	<0.0001
Delineation R2	2.83 ± 0.39	2.18 ± 0.71	<0.0001
Volume (mm^3^)	1505 ± 2303	1162 ± 1782	0.0010
CNR	48.1 ± 32.1	24.4 ± 15.3	<0.0001

Evaluations (in *n* = 40 patients) were performed separately for contrast-enhanced (CE) turbo field-echo (TFE) and Black Blood (BB) sequences as derived from intraoperative magnetic resonance imaging (iMRI) by two readers (R1 and R2).

## Abbreviations

BB	Black Blood
CE	Contrast-enhanced
CNR	Contrast-to-noise ratio
DES	Direct electrical stimulation
EOR	Extent of resection
HGG	High-grade glioma
iMRI	Intraoperative magnetic resonance imaging
MP-RAGE	Magnetization-prepared rapid gradient-echo
MRI	Magnetic resonance imaging
NAWM	Normal-appearing white matter
PACS	Picture Archiving and Communication System
PFS	Progression-free survival
R1	Reader 1
R2	Reader 2
ROI	Region of interest
SD	Standard deviation
SI	Signal intensity
TE	Echo time
TFE	Turbo field-echo
TR	Repetition time
TSE	Turbo spin-echo
WHO	World Health Organization
